# Ordinality: The importance of its trial list composition and examining its relation with adults’ arithmetic and mathematical reasoning

**DOI:** 10.1177/17470218211016794

**Published:** 2021-05-24

**Authors:** Helene Vos, Wim Gevers, Bert Reynvoet, Iro Xenidou-Dervou

**Affiliations:** 1 Department of Education and Pedagogy, Utrecht University, Utrecht, The Netherlands; 2 Research Unit Brain & Cognition, KU Leuven, Leuven, Belgium; 3 Centre for Research in Cognition and Neurosciences (CRCN), ULB Neurosciences Institute (UNI), Université Libre de Bruxelles, Brussels, Belgium; 4 Faculty of Psychology and Educational Sciences, KU Leuven, Kortrijk, Belgium; 5 Centre for Mathematical Cognition, Mathematics Education Centre, Loughborough University, Loughborough, UK

**Keywords:** Ordinality, arithmetic, mathematical reasoning, numerical cognition

## Abstract

Understanding whether a sequence is presented in an order or not (i.e., ordinality) is a robust predictor of adults’ arithmetic performance, but the mechanisms underlying this skill and its relationship with mathematics remain unclear. In this study, we examined (a) the cognitive strategies involved in ordinality inferred from behavioural effects observed in different types of sequences and (b) whether ordinality is also related to mathematical reasoning besides arithmetic. In Experiment 1, participants performed an arithmetic, a mathematical reasoning test, and an order task, which had balanced trials on the basis of order, direction, regularity, and distance. We observed standard distance effects (DEs) for ordered and non-ordered sequences, which suggest reliance on magnitude comparison strategies. This contradicts past studies that reported reversed distance effects (RDEs) for some types of sequences, which suggest reliance on retrieval strategies. Also, we found that ordinality predicted arithmetic but not mathematical reasoning when controlling for fluid intelligence. In Experiment 2, we investigated whether the aforementioned absence of RDEs was because of our trial list composition. Participants performed two order tasks: in both tasks, no RDE was found demonstrating the fragility of the RDE. In addition, results showed that the strategies used when processing ordinality were modulated by the trial list composition and presentation order of the tasks. Altogether, these findings reveal that ordinality is strongly related to arithmetic and that the strategies used when processing ordinality are highly dependent on the context in which the task is presented.

## Introduction

**Figure 1.**
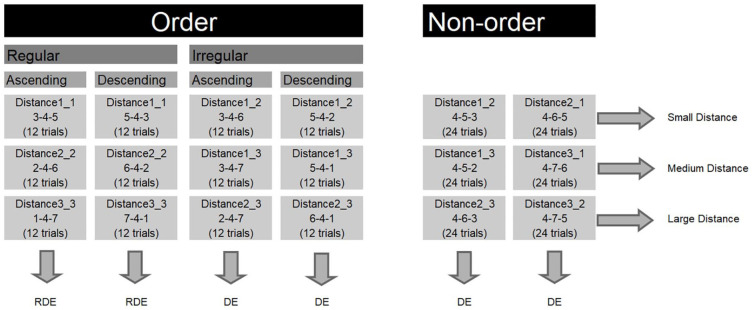
Conditions in the order task and the hypothesised effects for each condition. RDE: reversed distance effect; DE: distance effect. Distance1_1 implies that the distance between the first and the second digit was one and the distance between the second and the third digit was one. The displayed sequence is an example sequence for that specific condition. The number of trials in each condition is displayed between parentheses.

Elementary arithmetic comprises four basic operations, namely, addition, subtraction, multiplication, and division. These operations make it possible to handle various situations in daily life in which numbers play a role. In addition, arithmetic is also a building block for more complex mathematical skills. The domain of numerical cognition has intensively studied the predictors of arithmetic and mathematical skills. One predictor that has received increased attention in the past years is ordinality (for overviews, see Lyons et al., 2016; Sury & Rubinsten, 2012). Ordinality refers to the relation between items in a sequence and is often measured with an order task where different types of sequences are presented such as ordered sequences as 1-2-3 and non-ordered sequences as 3-1-2. In the order task, participants have to decide if a sequence is presented in an order or not. Results from previous studies have indicated that there is a strong relation between performance on the order task and arithmetic performance in both children (Attout & Majerus, 2017; Lyons & Ansari, 2015; Lyons et al., 2014; O’Connor et al., 2018, 2019; Sasanguie & Vos, 2018; Sommerauer et al., 2020; Vogel et al., 2015) and adults (Goffin & Ansari, 2016; Lyons & Beilock, 2009; Morsanyi et al., 2017; Orrantia et al., 2019; Sasanguie et al., 2017; Sella et al., 2020; Vogel et al., 2017, 2019; Vos et al., 2017). However, the mechanisms underlying this relationship remain relatively underspecified. Although studies have investigated how different types of sequences are processed (Lyons et al., 2016; Sury & Rubinsten, 2012), a systematic and balanced examination of the different types of sequences is still lacking (see Figure 1 for a more balanced example). More specifically, most studies have only included a specific set of sequences and there has often been an overrepresentation of a particular type of sequence. Before we assess the relation between ordinality and mathematical skills, we therefore first systematically examined the strategies involved when participants process ordinality. The aim of the current study was to, first, systematically unravel the strategies involved when processing different types of sequences in the order task and, second, to examine the relation between ordinality and mathematical skills.

Previous studies have shed light on the behavioural effects that occur when performing the order task and consequently provided interesting insights into the potential cognitive strategies that could be applied when processing different types of sequences (Franklin et al., 2009; Fulbright et al., 2003; Kaufmann et al., 2009; Lyons & Beilock, 2009, 2013; Lyons et al., 2014; Morsanyi et al., 2017; Vos et al., 2017). In general, the sequences presented in the order task can vary across four dimensions (Figure 1), and the strategy that is applied to process a sequence is dependent on these dimensions. The first dimension is *order* itself; sequences can be presented in an order (e.g., 3-4-5) or not (e.g., 3-5-4). This dimension has a strong influence on how a sequence is processed. In general, ordered sequences are processed faster than non-ordered sequences (Lyons & Beilock, 2009; Morsanyi et al., 2017; Orrantia et al., 2019; Vogel et al., 2017; Vos et al., 2017). However, this finding is strongly dependent on a second dimension: distance.

The second dimension is *distance*: the distance between the digits can be small (e.g., 3-4-5) or large (e.g., 1-4-9). A frequently observed effect found when processing ordered sequences is the reversed distance effect (RDE; Franklin et al., 2009; Goffin & Ansari, 2016; Lyons & Ansari, 2015; Lyons & Beilock, 2013): faster performance when the distance between the digits is small (e.g., 3-4-5 or 5-4-3) than when the distance between them is large (e.g., 1-4-7 or 7-4-1). Because the RDE is often observed for ordered sequences, it has been frequently considered as the hallmark of ordinality (Lyons & Ansari, 2015; Lyons & Beilock, 2013). The occurrence of the RDE can be attributed to different strategies that are used for small and large distance sequences (Vos et al., 2017). Sequences with a small distance between the digits (such as 1-2-3 and 3-2-1) are highly familiar and consist of digits that are strongly associated with each other. Consequently, these sequences can be easily retrieved from long-term memory. In contrast, sequences with a large distance between the digits are not familiar and the digits are less strongly associated with each other. For these sequences, decisions are probably based on magnitude comparison. More specifically, to decide whether a non-familiar sequence is presented in the correct order or not, the magnitude of the consecutive digits has to be assessed and compared. For instance, deciding whether the sequence 1-5-9 is in the correct order could involve two separate comparisons of the first pair and the last pair of digits (1-5 and 5-9, respectively). Thus, the RDE that is typically observed for ordered sequences is the result of fast retrieval from long-term memory for small distance sequences on one hand and the slower process of comparison for large distance sequences on the other.

While a RDE is observed for ordered sequences, a standard distance effect (DE) is observed for non-ordered sequences (Morsanyi et al., 2017; Vogel et al., 2017; Vos et al., 2017): slower performance when the distance between the digits is small than when the distance between the digits is large (e.g., 5-4-6 is processed slower than 6-4-7). The standard DE is considered standard because it has been consistently observed in comparison tasks where participants indicate the largest of two digits (Moyer & Landauer, 1967). The DE for non-ordered sequences occurs because both small and large non-ordered sequences are unfamiliar and consequently magnitude comparison is used for all non-ordered sequences.

Although most studies report a RDE for ordered sequences, some studies have found a DE instead (Turconi et al., 2004; Vogel et al., 2015). However, these studies only presented pairs of digits and could have therefore prompted comparison rather than order processing. Furthermore, while some studies report the RDE across several distances between the digits, other studies indicated that its appearance is dependent on the distances that are presented. For example, Turconi et al. (2006) presented pairs of digits and found a RDE only for distance one compared with other distances, but this was not found when comparing larger distances with each other. It could be argued that this was also due to the presentation of pairs of digits instead of triplets, but Goffin and Ansari (2016) and Vogel et al. (2017) found similar results in studies with triplets. Moreover, the way in which the DE is calculated varies across studies, which is a result of the distances that are presented in the order task. Some studies only included two distances and calculated the DE based on these two distances (Sasanguie et al., 2017; Sasanguie & Vos, 2018), while others have calculated the DE over three distances or more (Morsanyi et al., 2017; for example, the difference between distances one and two and two and three is calculated). Furthermore, some studies categorised distances in small distance trials in which the distance was one and large distance trials in which the distances were two, three, or more (Vos et al., 2017). In this case, distance one is overrepresented, and this could lead to a stronger RDE. To have a complete understanding of the role that distance plays in order processing and to see whether the DE is present across all distances, a balanced distribution of distance across trials is rendered necessary.

Within ordered sequences, two additional dimensions can be identified: regularity and direction. *Regularity* refers to the fact that sequences can be regular or irregular: in regular trials, the intervals between neighbouring digits are equal (e.g., 1-3-5), whereas in irregular trials, the intervals between neighbouring digits are unequal (e.g., 1-3-4). Most previous studies using the order task did not take regularity into account (Lyons & Ansari, 2015; Lyons & Beilock, 2013; Sasanguie et al., 2017; Vogel et al., 2017; Vos et al., 2017). Nevertheless, regularity has been shown to influence how an ordered sequence is processed. Lyons and Beilock (2009) found that participants processed regular ordered sequences of distance one faster than non-ordered sequences (e.g., 1-2-3 was processed faster than 2-1-3). The same was true for regular ordered sequences with a larger distance between the digits (e.g., 1-3-5 was processed faster than 5-1-3). In contrast, the processing of irregular ordered sequences did not differ from non-ordered sequences (e.g., there was no difference between the processing time of 1-2-8 and 1-8-2). This suggests that regular trials differ from irregular and non-ordered trials, but irregular ordered and non-ordered trials are processed similarly.

The fourth and last dimension is *direction*: sequences can be presented in an ascending (e.g., 3-4-5) or descending (e.g., 5-4-3) order. Studies typically observe a RDE for both ascending and descending sequences (Franklin et al., 2009; Franklin & Jonides, 2008; Vos et al., 2017). However, Vos et al. (2017) found that ascending sequences elicited faster performance than the descending ones, which could be due to stronger associations between the digits in the ascending sequences resulting in faster retrieval from memory. Furthermore, ascending sequences elicit a stronger RDE than the descending ones (Vos et al., 2017). An explanation for this could be that for ascending sequences, the difference in associations between small and large distance sequences is larger compared with descending sequences (e.g., there is a larger contrast in associative strength between the digits in the sequence 1-2-3 and 1-5-9 than between the digits in the sequences 3-2-1 and 9-5-1).

Altogether, the RDEs or DEs which have been observed in previous studies reflect different strategies that are used when processing ordinality, namely, retrieval from long-term memory and magnitude comparison. A possible explanation for the robust relation between ordinality and arithmetic is that both ordinality and arithmetic require retrieval from long-term memory and comparison. Retrieval from long-term memory and comparison play an important role in arithmetic performance (Campbell & Xue, 2001). As mentioned above and as indicated by several reviews and experimental studies, associative (or retrieval-based) strategies and comparison also play a role in ordinality (LeFevre & Bisanz, 1986; Lyons et al., 2016; Marshuetz & Smith, 2006; Rubinsten & Sury, 2011; Sasanguie et al., 2017; Sella et al., 2020; Sommerauer et al., 2020; Sury & Rubinsten, 2012; Vos et al., 2017). These strategies could potentially be driving the relation between ordinality and arithmetic performance.

Whereas numerous studies have found that ordinality is strongly related to arithmetic, the relation between ordinality and higher, more complex forms of mathematics such as mathematical reasoning has received little attention. While arithmetic only requires performing an operation (i.e., addition, subtraction, multiplication, or division) to calculate a numerical answer to a problem, mathematical reasoning requires one to think about the representation of a problem and to subsequently calculate an answer (Gilmore et al., 2018). Given the significant role that ordinality is assumed to play in arithmetic performance, it is an important step for both theory and practice to examine whether it is also related to mathematical reasoning. Recently, Morsanyi et al. (2018) observed that in adults, ordinality was related not only to arithmetic but also to mathematical reasoning which was assessed using a cognitive reflection task (measuring the ability to inhibit incorrect responses and instead rely on effortful processing) and a probabilistic reasoning scale (measuring the ability to reason statistically and make decisions about uncertain outcomes). In contrast, Orrantia et al. (2019) did not observe a relation between ordinality and a general mathematics achievement test where adults had to perform mental operations with numbers and quantitative concepts as fractions, percentages, and the base-10 system. In summary, research on the relation between ordinality and more complex mathematics measures is scarce, and findings have been inconclusive. Further research is needed to examine this relationship.

In the current study, we addressed several gaps in the literature about ordinality. While most previous studies have included the dimensions order and distance, only a few studies have included the dimensions direction (Vos et al., 2017) and regularity (Lyons & Beilock, 2009) in their assessment of ordinality. To the best of our knowledge, no study has included the dimensions order, distance, direction, and regularity concurrently in one experiment. Moreover, in most past studies, there was no balanced distribution of the different dimensions across the trials to systematically examine the mechanisms involved in ordinality. The current study aimed to further specify the mechanisms involved in ordinality and its relation to mathematics achievement by taking into account concurrently all four dimensions of ordinality and by assuring a balanced distribution of these dimensions. The first aim of the current study was to unravel the strategies involved when processing different types of sequences by systematically examining the behavioural effects observed for the different dimensions of the order task. Therefore, we administered an order task for which we balanced trials on the basis of order, distance, regularity, and direction (see Figure 1), and we examined systematically how each dimension is processed. In the presented order task, participants had to decide whether a sequence of three digits is presented in order or not. Half of the trials were ordered, the other half of the trials were non-ordered. Within the ordered trials, half of the trials were regular and half of the trials were irregular. Within the regular and irregular trials, half of the trials were ascending and half of the trials were descending. Each of the categories contained three distances, namely, small, medium, and large distances. The regular sequences included distances of one (small), two (medium), and three (large). For irregular sequences, the distance between neighbouring digits was one and two (small), one and three (medium), and two and three (large). For the non-ordered sequences, the following distances were presented: one and two (small), one and three (medium), and two and three (large). For half of the trials, the distance between the first and the second digit was larger than the distance between the second and the third digit; for the other half of the trials, it was the reverse. The second aim of the study was to investigate the well-established relation between ordinality and arithmetic and in addition also examine the relation between ordinality and more complex forms of mathematics, specifically mathematical reasoning.

## Experiment 1

The first experiment was pre-registered on AsPredicted. The pre-registered protocol is available at https://aspredicted.org/c675z.pdf. Participants performed an order task, an arithmetic test, and a mathematical reasoning test. To make sure the relation between the tasks was not the result of processing speed or fluid intelligence, we controlled for these two factors.

The sequences presented in the order task were manipulated across multiple dimensions. In this way, we aimed to gain more insight into the strategies involved in the processing of different types of sequences. Both order (e.g., order and non-order) and distance (e.g., small, medium, and large distances) were manipulated. Also, within the ordered sequences, there was a manipulation of direction (i.e., ascending and descending) and regularity (i.e., regular and irregular). We hypothesised that a comparison strategy can be applied to all types of sequences but can be bypassed by fact retrieval when there are strong associations between the items in a sequence (e.g., 3-4-5). Figure 1 illustrates the behavioural effects that were hypothesised for each condition based on this hypothesis. In regular sequences with a small distance between the digits, digits are highly associated with each other and can be easily retrieved from long-term memory, while for regular sequences with a large distance, probably comparison is used. Therefore, we predicted a RDE for regular sequences. On the contrary, in irregular and non-ordered sequences, digits in both the small and large distance sequences are not strongly associated and will probably be processed by multiple digit comparison. Hence, we predicted a DE for these sequences.

With regard to the relation between ordinality and mathematics performance, we hypothesised that ordinality would explain variance in arithmetic above and beyond processing speed and fluid intelligence because both ordinality and arithmetic require comparison and fact retrieval. However, because mathematical reasoning involves mostly abstract reasoning, we expected that ordinality would not predict mathematical reasoning above processing speed and fluid intelligence.

### Method

#### Participants

As our pre-registration indicates, we recruited and tested 60 participants at Loughborough University (*M*_age_ = 23.07 years, *SD*_age_ = 6.74; 28 men, 32 women). Of these participants, 65% had a British citizenship (of which 3.33% had a dual citizenship), the rest had a citizenship from another country. All participants spoke English. As pre-registered, we excluded participants pairwise from the analysis for a task when their standardised *z*-score was greater than 2.58 on a task. This resulted in the removal of the scores of five participants on one of the experimental tasks (we excluded three participants for the order task, one participant for the mathematical reasoning test, and two participants for the processing speed task). Subsequently, the repeated-measures analysis of variance (ANOVA) for the performance on the order task included 57 participants. The hierarchical regression analysis with arithmetic performance as dependent variable included 55 participants, and the hierarchical regression analysis with mathematical reasoning included 54 participants.

#### Procedure

The study was approved by the Ethics Committee of Loughborough University. Before the start of the experiment, participants received information about the general nature of the procedure and subsequently signed an informed consent. The administration of the experimental tasks took place in the following order: Tempo Test Arithmetic, processing speed task, Wechsler Individual Achievement Test–II (WIAT-II) mathematical reasoning test, order task, and a short form of Raven’s Advanced Progressive Matrices (Raven’s APM).

Participants performed the Tempo Test Arithmetic and the short form of Raven’s APM by paper and pencil. For the WIAT-II mathematical reasoning test, the experimenter scored oral responses of the participants. The order task and processing speed task were presented in E-Prime Professional software, Version 3.0 (Psychological Software Tools, Pittsburgh, PA, USA), on a 15-inch colour screen laptop with a QWERTY keyboard.

#### Tasks

##### Order task

In the order task, every trial started with a fixation cross of 600 ms. Subsequently, a triplet of single digits (range: 1–9) appeared on the screen for 1,000 ms after which a blank screen appeared. During the stimulus presentation or during the blank screen, participants had to indicate whether the sequence was presented in order by pressing “q” if the sequence was ordered (either ascending or descending) and by pressing “p” if the sequence was non-ordered. After a response was given, an intertrial interval of 1,500 ms followed.

The task consisted of 288 trials. The number of trials in each condition is displayed in Figure 1. Both accuracies and median reaction times for correct responses on this task were used as an index of the performance on the order task.

##### Standardised arithmetic test

Arithmetic performance was measured by an adapted version of the Tempo Test Arithmetic (De Vos, 1992; Guillaume et al., 2016). The Tempo Test Arithmetic is a time-limited test, which consists of five subtests: addition, subtraction, multiplication, division, and mixed operations. Every subtest consists of 50 items presented in increasing difficulty. Participants had 1 minute to solve as many problems as possible of each subtest. For each correct item, one point was credited. The raw score of the number of correct responses was used as an index of arithmetic performance.

##### Mathematical reasoning test

Mathematical reasoning was measured with the mathematical reasoning subtest of the Wechsler Individual Achievement Test (WIAT; Wechsler, 2005). This test presents a series of problems with both verbal and visual prompts requiring counting, identifying geometric shapes and patterns, interpreting graphs, and solving multiple step word problems. The test presents questions with regard to time, money, measurement, statistics, and probability. The numbers used in the text could be either whole numbers, fractions, or decimals. The raw score of the number of correct responses was used as an index of mathematical reasoning.

##### Processing speed task

Participants performed a processing speed task to control for general processing speed (see Reigosa-Crespo et al., 2011, for a similar task). Participants had to press the space bar as soon as a black square appeared on the screen. After the response, the square disappeared, followed by an inter-stimulus presentation time varying between 500 and 1,500 ms. The task started with the presentation of four practice trials followed by 20 test trials. The median RT on this task was taken into account as an index of processing speed.

##### Raven’s APM (short form)

A short form of Raven’s APM (Arthur & Day, 1994) was administered to measure general fluid intelligence. The task administration started with two practice items from Raven’s Standard Progressive Matrices (SPM; Raven, 1938). The test items were 12 items from Raven’s APM. The raw score of the number of correct responses was used as an index of fluid intelligence.

### Results

The following main analyses were pre-registered. First, to examine the behavioural effects (i.e., DEs and RDEs) of the different sequences in the order task, we conducted repeated-measures ANOVAs. Second, we analysed whether ordinality predicted arithmetic performance and mathematical reasoning. Median reaction times were analysed because they are less sensitive to a skewed distribution (Whelan, 2008). In addition to the main analyses, we also pre-registered several sub-analyses. To examine which dimensions in the order task uniquely predicted the median RT on the task, we conducted a by-item linear regression with the dimensions of the trials in the order task as predictors and the median reaction time for correct responses as dependent variable (see Supplementary Material A). Secondly, we pre-registered that the main focus of our analyses was on the median reaction times, but we also conducted the analyses for accuracy scores (see Supplementary Material B).

#### Pre-registered analyses

##### The effects of order, distance, regularity, and direction

Tables 1 and 2 present median reaction times for correct responses in each condition and the mean accuracies for each condition. First, we analysed the median RT for correct responses in both ordered and non-ordered trials. Second, because ordered trials can also be distinguished regarding regularity and direction, we analysed the median RT for correct responses of the ordered trials separately. Table 3 displays for which sequences RDEs and DEs were found.

**Table 1.** Mean accuracies (proportion), median reaction times (RTs in milliseconds), and the corresponding standard deviations per condition for ordered and non-ordered sequences of the order task.

	Accuracy	RT
Order small	.86 (.14)	1,094 (370)
Order medium	.88 (.12)	1,085 (380)
Order large	.90 (.13)	1,040 (356)
Non-order small	.90 (.09)	1,234 (421)
Non-order medium	.93 (.07)	1,165 (396)
Non-order large	.95 (.07)	1,137 (390)

**Table 2.** Mean accuracies (proportion), median reaction times (RTs in milliseconds), and the corresponding standard deviations per condition for ordered sequences of the order task.

	Accuracy	RT
Ascending regular small	.93 (.10)	1,050 (590)
Ascending regular medium	.94 (.07)	1,002 (357)
Ascending regular large	.95 (.07)	958 (302)
Ascending irregular small	.87 (.22)	1,182 (479)
Ascending irregular medium	.85 (.22)	1,176 (492)
Ascending irregular large	.89 (.17)	1,112 (443)
Descending regular small	.87 (.17)	1,137 (406)
Descending regular medium	.88 (.16)	1,093 (371)
Descending regular large	.87 (.17)	1,030 (413)
Descending irregular small	.79 (.24)	1,150 (397)
Descending irregular medium	.85 (.21)	1,099 (429)
Descending irregular large	.87 (.21)	1,078 (339)

**Table 3.** Occurrence of RDEs and DEs for the different type of sequences.

	Order				Non-order
	Regular		Irregular		
	Ascending	Descending	Ascending	Descending	
Hypothesised effect	RDE	RDE	DE	DE	DE
RDE	−	−	−	−	−
DE	*	−	*	−	*

RDE: reversed distance effect; DE: distance effect.

The table indicates whether an effect was absent or present for each separate condition. A dash (–) indicates that the effect was absent. An asterisk (*) indicates that the effect was present.

To examine the influence of order and distance on the median reaction times for correct responses, we conducted a 2 (order: order, non-order)^1^ × 3 (distance: small, medium, large) repeated-measures ANOVA. In the case of violation of the assumption of sphericity, degrees of freedom were corrected using Greenhouse–Geisser estimates. Results showed a significant effect of order, *F*(1, 56) = 28.84, *p* < .001, $\eta_{p}^{2}$ = .34. Ordered sequences (1,073 ms) were processed faster than non-ordered sequences (1,179 ms). In addition, a significant effect of distance was observed, *F*(1.42, 79.75) = 13.50, *p* < .001, $\eta_{p}^{2}$ = .19. Sequences with a small distance (1,164 ms) were processed slower than sequences with a medium distance (1,125 ms), and sequences with a medium distance were processed slower than sequences with a large distance (1,089 ms). Finally, no interaction between order and distance was found, *F*(2, 112) = 2.52, *p* = .085, $\eta_{p}^{2}$ = .04.

For the ordered sequences, we investigated the influence of direction, regularity, and distance on the median RT for correct responses, by a 2 (direction: ascending, descending) × 2 (regularity: regular, irregular) × 3 (distance: small, medium, large) repeated-measures ANOVA. There was a main effect of distance, *F*(2, 106) = 14.20, *p* < .001, $\eta_{p}^{2}$ = .21. Sequences with a small distance (1,130 ms) were processed slower than sequences with a medium distance (1,093 ms), and sequences with a medium distance were processed slower than sequences with a large distance (1,044 ms). A main effect of regularity was found, *F*(1, 53) = 26.92, *p* < .001, $\eta_{p}^{2}$ = .34. Regular trials (1,045 ms) were processed faster than irregular trials (1,133 ms). However, there was no main effect of direction, *F*(1, 53) = 0.56, *p* = .457, $\eta_{p}^{2}$ = .01. The interaction between direction and regularity was significant, *F*(1, 53) = 35.71, *p* < .001, $\eta_{p}^{2}$ = .40. For ascending sequences, there was a significant difference between regular (1,003 ms) and irregular sequences (1,157 ms), *F*(1, 54) = 44.58, *p* < .001, $\eta_{p}^{2}$ = .45. For descending sequences, there was no difference between regular (1,087 ms) and irregular sequences (1,109 ms), *F*(1, 53) = 1.73, *p* = .195, $\eta_{p}^{2}$ = .03. For regular sequences, ascending sequences were processed faster (1,003 ms) than descending sequences (1,087 ms), *F*(1, 56) = 12.55, *p* = .001, $\eta_{p}^{2}$ = .19. For irregular sequences, there was no difference between ascending sequences (1,157 ms) and descending sequences (1,109 ms), *F*(1, 53) = 3.36, *p* = .073, $\eta_{p}^{2}$ = .06. Finally, no interaction effects were observed with distance.

##### Relation between ordinality and arithmetic performance

Table 4 displays the zero-order correlations between the experimental tasks and shows a relation between ordinality median RT for correct responses and arithmetic. To examine whether the median reaction time on the order task explained unique variance in arithmetic performance, a hierarchical regression analysis was conducted with arithmetic as the dependent variable (Table 5). We included processing speed and fluid intelligence in the first step followed by a second step including the median RT on the order task. Collinearity between the tasks imposed no problem, because all variance inflation factors (VIFs) were ⩽1.01 and therefore within an acceptable range (Field, 2009). Processing speed and fluid intelligence explained about 7% of the variance in arithmetic performance which was not significant. The median RT on the order task explained about 29% additional variance and contributed significantly to arithmetic performance.

**Table 4.** Bivariate correlations between the experimental tasks.

		1	2	3	4	5
1	Ordinality Accuracy					
2	Ordinality RT	−.07				
3	Arithmetic	.17	−.58**			
4	Mathematical reasoning	.32*	−.20	.24		
5	Fluid intelligence	.46**	−.17	.10	.46**	
6	Processing speed	−.01	−.12	.24	−.15	−.12

* *p* < .05, **p < .01.

**Table 5.** Hierarchical regression with arithmetic as dependent variable.

Step	Independent variables	Standardised β	*t*	*p*	*R* ^2^	Δ*R*^2^
1	Processing speed	.24	1.80	.078	.07	.07
	Fluid intelligence	.13	0.93	.357		
2	Processing speed	.17	1.47	.149	.36**	.29**
	Fluid intelligence	.02	0.21	.832		
	Ordinality RT	−.56	−.4.85	<.001		

** *p* < .01.

#### Exploratory analysis

##### Relation between ordinality and mathematical reasoning

As shown in Table 4, there was no relation between the median RT for correct responses on the order task and mathematical reasoning. Although there was no relation between the median RT for correct responses on the order task and mathematical reasoning, the accuracy on the order task was related to mathematical reasoning. In addition, there was a correlation between fluid intelligence and mathematical reasoning. As an exploratory analysis, we conducted a hierarchical regression analysis with mathematical reasoning as the dependent variable (Table 6). We included fluid intelligence and processing speed in the first step of the analysis followed by a second step including the accuracy on the order task (in contrast to our pre-registration where only reaction time was pre-registered as dependent variable). All VIFs were within an acceptable range and ⩽1.25 (Field, 2009). Fluid intelligence significantly explained about 23% of the variance in mathematical reasoning. Including the accuracy on the order task as an additional predictor explained about 1% additional variance, which was not significant.

**Table 6.** Hierarchical regression with mathematical reasoning as dependent variable.

Step	Independent variables	Standardised β	*t*	*p*	*R* ^2^	Δ*R*^2^
1	Processing speed	−.08	−0.62	.535	.23**	.23**
	Fluid intelligence	.47	3.83	<.001		
2	Processing speed	−.08	−0.66	.512	.24**	.01
	Fluid intelligence	.42	2.96	.005		
	Ordinality Accuracy	.10	0.69	.491		

** *p* < .01.

### Discussion

In the first experiment, we found a relation between ordinality and individual differences in arithmetic performance which is in line with previous findings (Goffin & Ansari, 2016; Lyons & Beilock, 2009, 2013; Lyons et al., 2014; Morsanyi et al., 2017; Sasanguie et al., 2017; Vogel et al., 2017; Vos et al., 2017). In addition, a relation between mathematical reasoning and ordinality was observed, but this relation was completely explained away by fluid intelligence. The strong relation between ordinality and arithmetic performance might be a result of similar strategies, which are applied when processing ordinality and when performing arithmetic. Depending on the sequence that is presented, participants use different strategies such as retrieval and comparison to decide whether the sequence is ordered or not. Similarly, different arithmetic operations require different strategies (e.g., fact retrieval or procedural strategies). Furthermore, the strong relation between ordinality and arithmetic can possibly be attributed to the ability of flexibly choosing and using different strategies when processing order and when performing arithmetic.

By examining the behavioural effects in the order task, we could infer which strategies participants were relying on while processing different types of sequences. In line with our hypothesis, there was a DE for non-ordered sequences, a finding that has been observed in several previous studies (Lyons & Ansari, 2015; Vos et al., 2017). Furthermore, as hypothesised and in line with previous findings (Lyons & Beilock, 2009), a DE for ordered irregular sequences was found. This DE observed for both irregular ordered and non-ordered sequences suggests that these sequences are processed similarly and that participants rely on comparison strategies probably because the associations between the digits in these sequences are weak. Surprisingly, though, there was also a DE for ordered regular sequences. This finding is in contrast with previous studies that found a RDE for ordered regular sequences (Goffin & Ansari, 2016; Lyons & Ansari, 2015; Lyons & Beilock, 2013; Morsanyi et al., 2017; Sasanguie et al., 2017; Vos et al., 2017). The typically observed RDE indicated that participants retrieved ordered regular small distance sequences from memory but used more time-consuming strategies as digit comparison for ordered regular large distance sequences. In contrast, the DE observed in the current experiment suggests that participants relied on comparison strategies for both ordered regular small and large distance sequences.

A reason for the different results in the current study compared with previous studies could be that in the current study, there were less trials that elicited retrieval strategies due to several manipulations. First, while many previous studies have only included ordered trials that were regular (Lyons & Ansari, 2015; Lyons & Beilock, 2013; Sasanguie et al., 2017; Vogel et al., 2017; Vos et al., 2017), we also took into account irregular trials that trigger comparison strategies for both small and large distance sequences resulting in a DE. Second, trials with distance one (i.e., trials that trigger a retrieval strategy) were less frequent compared with previous studies (e.g., Vos et al., 2017, only contrasted distance one with distances two, three, and four). Third, trials with the strongest associations (e.g., 1-2-3 and 2-3-4) were not presented to make sure that the overall distance was balanced for the regular, irregular, and non-ordered conditions. In sum, while in other studies participants might have anticipated a retrieval strategy, a reduction of trials that elicit retrieval strategies might have resulted in a more frequent use of comparison strategies in our study. Consequently, comparison may have even been applied to those trials that could be solved by retrieval and this resulted in a DE. Thus, the trial list composition list might affect the presence or absence of the RDE.

In the second experiment, we further tested whether the increased exposure to trials requiring comparison strategies and decreased exposure to trials requiring retrieval strategies may explain the absence of a RDE in the first experiment. In addition, we again examined the relation between ordinality and arithmetic, and we investigated whether performance on specific sequences correlated more strongly with certain arithmetic operations.

## Experiment 2

**Figure 2.**
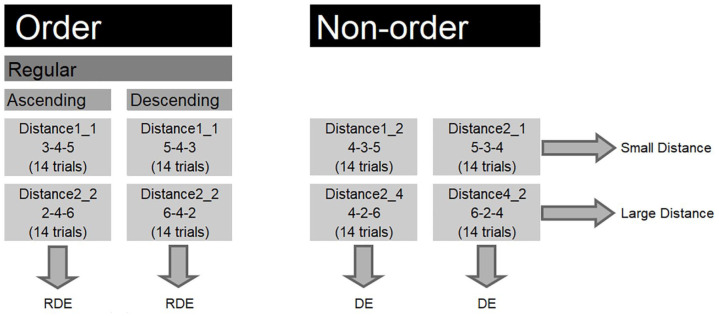
Conditions in the common order task and the hypothesised effects for each condition. RDE: reversed distance effect; DE: distance effect. The displayed sequence is an example sequence for that specific condition. The number of trials in each condition is displayed between parentheses.

**Figure 3.**
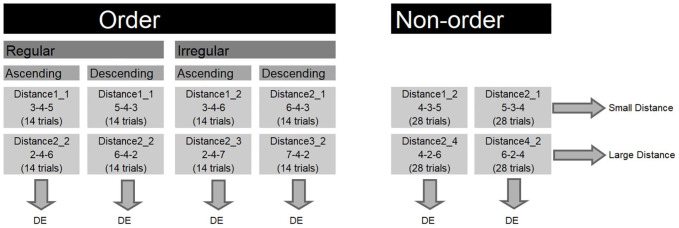
Conditions in the balanced order task and the hypothesised effects for each condition. DE: distance effect. The displayed sequence is an example sequence for that specific condition. The number of trials in each condition is displayed between parentheses.

The second experiment was also pre-registered on AsPredicted. The pre-registered protocol is available at https://aspredicted.org/ed4br.pdf. Participants performed two digit order tasks that differed with respect to the presented sequences. In the first digit order task, regular ordered sequences and non-ordered sequences were included. We will refer to this task as the common order task, because order tasks with regular and non-ordered sequences (but not irregular sequences) are most commonly used in the literature (Lyons & Ansari, 2015; Lyons & Beilock, 2013; Sasanguie et al., 2017; Vogel et al., 2017; Vos et al., 2017). For this task, half of the ordered trials in the trial list were expected to trigger retrieval from long-term memory and half of the ordered trials in the trial list were expected to trigger comparison strategies. Because retrieval and comparison strategies are triggered to the same extent in the ordered trials, we expected that participants would use both retrieval and comparison strategies when performing the common order task. Figure 2 illustrates the behavioural effects that were hypothesised for each condition. In this task, we predicted a RDE for ordered sequences but a DE for non-ordered sequences. In the second order task (balanced order task), regular, irregular, and non-ordered sequences were included. This task is similar to the task that was presented in Experiment 1 and resembles the tasks in some previous studies (Lyons & Beilock, 2009; Orrantia et al., 2019). We will refer to this order task as the balanced order task because regular and irregular trials were represented equally among the ordered trials. For this task, one fourth of the ordered trials in the trial list were expected to trigger retrieval strategies, while the rest of the ordered trials in the trial list was expected to trigger comparison strategies. Here, we expected that the majority of the ordered trials would trigger comparison strategies and that participants would consequently start to apply comparison to all the sequences. Hence, we expected DEs for all the sequences (see Figure 3). Finally, we further investigated the relation between ordinality and arithmetic performance by examining the relation between specific sequences in the two order tasks and certain arithmetic operations (i.e., arithmetic operations requiring retrieval such as single digit addition and multiplication and arithmetic operations requiring procedural strategies such as multiple digit subtraction).

### Method

#### Participants

As pre-registered, 60 Dutch-speaking participants from the University of Leuven took part in the current study for course requirements (*M*_age_ = 19.05 years, *SD* = 3.50; 37 women). As pre-registered, we excluded participants pairwise from the analysis for a task when their standardised *z*-score was greater than 2.58 on a task. This resulted in removing the scores of 14 participants on one of the experimental tasks (we excluded two participants for the common order task, three participants for the balanced order task, two participants for the Tempo Test Arithmetic, three participants for the single digit addition task, three participants for the multiple digit subtraction task, five participants for the single digit multiplication task, and three participants for the processing speed task). Subsequently, the analysis for the performance on the common order task included 58 participants. The analysis for the performance on the balanced order task included 57 participants. The analysis on both order tasks included 56 participants. The analyses on all the tasks included 45 participants.

#### Procedure

The study was approved by the Ethics Committee of the University of Leuven. At the start of the experiment, participants received information about the procedure of the experiment after which they signed an informed consent. For half of the participants, the experimental tasks were presented in the following order: Tempo Test Arithmetic, common order task, single digit addition, multiple digit subtraction, single digit multiplication, balanced order task, and processing speed task. The other half of the participants performed the experimental tasks in the same order, except that the two order tasks were presented in reversed order (i.e., the balanced order task was administered before the common order task). The Tempo Test Arithmetic was administered by paper and pencil. The other tasks were presented in E-Prime Professional software, Version 3.0 (Psychological Software Tools), on a 15-inch colour screen laptop with an AZERTY keyboard.

#### Tasks

##### Order tasks

In the order tasks, the presentation time of the fixation cross, presented triplet of single digits, blank screen, and intertrial interval was similar as in Experiment 1 . The tasks differed with regard to the trial list composition as is displayed in Figures 2 and 3. The common order task consisted of 112 trials. The balanced order task consisted of 224 trials. Both accuracies and median reaction times for correct responses on these tasks were used as an index of the performance on the order tasks.

##### Standardised arithmetic test

Similar as in Experiment 1, an adapted version of the Tempo Test Arithmetic (De Vos, 1992) was used to assess arithmetic performance (Guillaume et al., 2016). However, this time, in line with the arithmetic verification tasks, only the addition, subtraction, and multiplication problems were assessed.

##### Arithmetic verification tasks

In the arithmetic verification tasks, participants had to verify a series of horizontally presented arithmetic calculations. In the verification tasks, each trial started with a fixation cross of 600 ms after which an arithmetic problem with an outcome was presented on the screen. After the participants responded, there was an intertrial interval of 1,500 ms. Participants were instructed to press “a” when the arithmetic problem was presented with a correct answer and to press “p” when the arithmetic problem was presented with an incorrect answer. Arithmetic verification tasks with the following types of calculations were presented: single digit addition, multiple digit subtraction, and single digit multiplication. Median RTs for correct responses were used as an index of the arithmetic verification tasks.

*Single digit addition*: In the verification task with single digit addition, 48 trials^2^ were administered. Twelve unique items were presented; these were items with operands from 1 to 4 when ties are excluded (see Barrouillet & Thevenot, 2013). These trials were presented two times with the correct answer, resulting in 24 trials where the presented addition was correct. Furthermore, the trials were presented two times with an incorrect answer: one time with the correct answer minus 1 and one time with the correct answer plus 1. This resulted in 24 trials where the presented addition was incorrect.

*Multiple digit subtraction*: In the verification task with multiple digit subtraction, 48 trials were administered. Twelve unique items were presented. Half of these items required carrying, half of the items did not. The unique trials were presented two times with the correct answer, which resulted in 24 trials where the presented subtraction was correct. In addition, the unique trials were presented two times with an incorrect answer resulting in 24 trials where the presented subtraction was incorrect.

*Single digit multiplication*: In the verification task with single digit multiplication, 48 trials were administered. Twelve unique items were presented, that is, items with operands from 1 to 4 when ties are excluded. These unique trials were presented two times with the correct answer, resulting in 24 trials where the presented multiplication was correct. In addition, the unique trials were presented two times with an incorrect table-related answer, resulting in 24 trials where the presented multiplication was incorrect.

##### Processing speed task

Participants performed a processing speed task to control for general processing speed. In contrast to Experiment 1 where participants merely had to respond to a visual stimulus, this processing speed task required participants to actively process whether three presented digits were odd or even. In this task, each trial started with a fixation cross of 600 ms after which three single digits (range: 1–9) were presented on the screen for 1,000 ms. Consequently, a blank screen appeared. The task started with the presentation of four practice trials followed by 24 test trials. For half of the trials, all the digits in the sequence were odd, and for the other half of the trials, all the digits presented in the sequence were even. Participants were instructed to press “a” when the three presented digits were odd and to press “p” when the digits were even. Participants could respond during stimulus presentation or during the blank screen. After the participants responded, there was an intertrial interval of 1,500 ms. The median RT for correct responses on this task was taken into account as an index of processing speed.

### Results

The following main analyses were pre-registered. First, we analysed the behavioural effects (i.e., DEs and RDEs) of the different sequences in the common order task and the balanced order task with repeated-measures ANOVAs. Second, we computed partial correlations between the different sequences of each order task and, respectively, the Tempo Test Arithmetic, single digit addition, single digit multiplication, and multiple digit subtraction when controlling for processing speed, and we compared these correlations with Hotelling–Williams tests. Besides these pre-registered main analyses, the sub-analyses for accuracies were preregistered and these analyses are reported in the Supplementary Material (see Supplementary Material C). Additionally, the Supplementary Material contains an elaborate description of the repeated-measures ANOVA including presentation order (see Supplementary Material D).

#### Pre-registered analyses

##### Common order task

Tables 7 and 8 present median reaction times for correct responses and the mean accuracies for each condition of the common order task. Table 9 displays for which sequences RDEs and DEs were found. The influence of order and distance on the median RT for correct responses was examined by conducting a 2 (order: order, non-order) × 2 (distance: small, large) repeated-measures ANOVA. Results indicated a main effect of order, *F*(1, 57) = 19.51, *p* < .001, $\eta_{p}^{2}$ = .26, and distance, *F*(1, 57) = 11.35, *p* = .001, $\eta_{p}^{2}$ = .17. Ordered sequences (1,055 ms) were processed faster compared with non-ordered sequences (1,158 ms), and small distance sequences (1,135 ms) were processed slower than large distance sequences (1078ms). Furthermore, an interaction between order and distance was found, *F*(1, 57) = 13.65, *p* < .001, $\eta_{p}^{2}$ = .19. While no DE was found for ordered sequences, *t*(57) = 0.22, *p* = .826, a standard DE was found for non-ordered sequences, *t*(57) = 4.69, *p* < .001.

**Table 7.** Mean accuracies (proportion), median reaction times (RTs in milliseconds), and the corresponding standard deviations per condition for ordered sequences and non-ordered sequences of the common order task.

	Accuracy	RT
Order small	.93 (.05)	1,057 (332)
Order large	.92 (.07)	1,052 (335)
Non-order small	.92 (.06)	1,212 (406)
Non-order large	.95 (.07)	1,104 (394)

**Table 8.** Mean accuracies (proportion), median reaction times (RTs in milliseconds), and the corresponding standard deviations per condition for ordered sequences of the common order task.

	Accuracy	RT
Ascending small	.96 (.06)	996 (321)
Ascending large	.96 (.06)	1,023 (344)
Descending small	.91 (.08)	1,124 (376)
Descending large	.89 (.11)	1,115 (355)

**Table 9.** Occurrence of RDEs and DEs for the different types of sequences of the common order task.

	Order		Non-order
	Regular		
	Ascending	Descending	
Hypothesised effect	RDE	RDE	DE
RDE	−	−	−
DE	−	−	*

RDE: reversed distance effect; DE: distance effect.

The table indicates whether an effect was absent or present for each separate condition A dash (–) indicates that the effect was absent. An asterisk (*) indicates that the effect was present.

For the ordered trials, we examined the influence of direction and distance on the median RT for correct responses by a 2 (direction: ascending, descending) × 2 (distance: small, large) repeated-measures ANOVA. An effect of direction was observed, *F*(1, 57) = 52.53, *p* < .001, $\eta_{p}^{2}$ = .48. Ascending sequences (1,010 ms) were processed faster than descending sequences (1,120 ms). However, results indicated no effect of distance, *F*(1, 57) = 0.17, *p* = .680, $\eta_{p}^{2}$ = .003, and no interaction between direction and distance, *F*(1, 57) = 1.34, *p* = .251, $\eta_{p}^{2}$ = .02.

##### Balanced order task

Tables 10 and 11 present median reaction times for correct responses and the mean accuracies for each condition of the balanced order task. Table 12 displays for which sequences RDEs and DEs were found. The influence of order and distance on the median RT for correct responses was investigated by conducting a 2 (order: order, non-order) × 2 (distance: small, large) repeated-measures ANOVA. No main effects were present for order, *F*(1, 56) = 1.83, *p* = .181, $\eta_{p}^{2}$ = .03, and distance, *F*(1, 56) = 0.24, *p* = .626, $\eta_{p}^{2}$ = .004. Similarly, no interaction between order and distance was observed, *F*(1, 56) = 0.80, *p* = .375, $\eta_{p}^{2}$ = .01.

**Table 10.** Mean accuracies (proportion), median reaction times (RTs in milliseconds), and the corresponding standard deviations per condition for ordered sequences and non-ordered sequences of the balanced order task.

	Accuracy	RT
Order small	.78 (.14)	1,256 (323)
Order large	.81 (.13)	1,261 (375)
Non-order small	.80 (.13)	1,290 (335)
Non-order large	.82 (.12)	1,270 (346)

**Table 11.** Mean accuracies (proportion), median RT (RTs in milliseconds), and the corresponding standard deviations per condition for ordered sequences of the balanced order task.

	Accuracy	RT
Ascending regular small	.82 (.15)	1,219 (314)
Ascending regular large	.83 (.12)	1,214 (369)
Ascending irregular small	.78 (.20)	1,249 (317)
Ascending irregular large	.81 (.19)	1,272 (382)
Descending regular small	.78 (.15)	1,292 (382)
Descending regular large	.82 (.14)	1,234 (321)
Descending irregular small	.74 (.23)	1,296 (337)
Descending irregular large	.79 (.18)	1,286 (329)

**Table 12.** Occurrence of RDEs and DEs for the different type of sequences of the balanced order task.

	Order				Non-order
	Regular		Irregular		
	Ascending	Descending	Ascending	Descending	
Hypothesised effect	DE	DE	DE	DE	DE
RDE	−	−	−	−	−
DE	−	−	−	−	−

RDE: reversed distance effect; DE: distance effect.

The table indicates whether an effect was absent or present for each separate condition A dash (–) indicates that the effect was absent.

For the ordered trials, we examined the influence of direction, regularity, and distance on the median RT for correct responses by a 2 (direction: ascending, descending) × 2 (regularity: regular, irregular) × 2 (distance: small, large) repeated-measures ANOVA. Effects were observed for both direction, *F*(1, 55) = 4.36, *p* = .041, $\eta_{p}^{2}$ = .07, and regularity, *F*(1, 55) = 8.50, *p* = .005, $\eta_{p}^{2}$ = .13. Ascending sequences (1,238 ms) were processed faster than descending sequences (1,277 ms), and regular sequences (1,240 ms) were processed faster than irregular sequences (1,276 ms). However, there was no effect of distance, *F*(1, 55) = 0.39, *p* = .533, $\eta_{p}^{2}$ = .01. Furthermore, no interaction effects were found.

##### Relation between ordinality and arithmetic calculations

Table 13 displays the partial correlations between the median reaction times for correct responses on the sequences presented in the order tasks, Tempo Test Arithmetic, single digit addition, multiple digit subtraction, and single digit multiplication after controlling for processing speed. Consequently, we conducted Hotelling–Williams tests to examine (a) whether the correlations differed significantly across the arithmetic operations (see Table 14) and (b) whether the correlations between the sequences and the arithmetic operations differed between the two tasks (see Table 15). In total, 36 Hotelling–Williams tests were performed. To correct for the problem of multiple comparisons, we tested each hypothesis at a significance level of .001 (α = .05/36 = .001). Results indicated no significant difference between the correlations.

**Table 13.** Partial correlations between the Tempo Test Arithmetic, the median reaction times on the conditions of the common and balanced order task, and the median reaction times on the arithmetic verification tasks after controlling for processing speed.

		1	2	3	4	5	6	7	8	9	10	11
1	Tempo Test Arithmetic											
2	Addition	−.37*										
3	Subtraction	−.61**	.65**									
4	Multiplication	−.48**	.60**	.62**								
5	Common order task—Order small	.05	.33*	.22	.22							
6	Common order task—Order large	.13	.21	.10	.10	.92**						
7	Common order task—Non-order small	.05	.36*	.18	.13	.87**	.83**					
8	Common order task—Non-order large	.11	.22	.11	.02	.80**	.82**	.90**				
9	Balanced order task—Order small	−.25	.53**	.53**	.53**	.53**	.45**	.44**	.32*			
10	Balanced order task—Order large	−.17	.44**	.40**	.40**	.47**	.51**	.44**	.30	.79**		
11	Balanced order task—Non-order small	−.27	.61**	.54**	.49**	.58**	.49**	.57**	.42**	.89**	.79**	
12	Balanced order task—Non-order large	−.26	.60**	.50**	.44**	.54**	.48**	.52**	.36*	.91**	.85**	.95**

* *p* < .05, **p < .01.

**Table 14.** Hotelling–Williams tests for the difference in correlations between the median reaction times on the conditions of each order task and the arithmetic operations after controlling for processing speed.

Task	Sequence	Addition vs. subtraction		Addition vs. multiplication		Subtraction vs. multiplication	
		*t*	*p*	*t*	*p*	*t*	*p*
Common order task	Order small	0.87	.390	0.81	.422	0	1.00
	Order large	0.84	.406	0.79	.437	0	1.00
	Non-order small	1.44	.157	1.73	.092	0.36	.718
	Non-order large	0.84	.405	1.45	.156	0.65	.520
Balanced order task	Order small	0	1.00	0	1.00	0	1.00
	Order large	0.34	.739	0.32	.754	0	1.00
	Non-order small	0.67	.505	1.07	.293	0.43	.667
	Non-order large	0.94	.353	1.39	.171	0.50	.618

**Table 15.** Hotelling–Williams tests for the difference between the order tasks regarding the correlations between the sequences and arithmetic operations after controlling for processing speed.

	Addition		Subtraction		Multiplication	
	*t*	*p*	*t*	*p*	*t*	*p*
Order small	−1.51	.139	−2.35	.024	−2.35	.024
Order large	−1.61	.116	−2.08	.044	−2.08	.044
Non-order small	−2.10	.041	−2.92	.006	−2.83	.007
Non-order large	−2.57	.014	−2.47	.018	−2.60	.013

#### Exploratory analyses

##### Modulation of task

To test whether the observed effects were modulated by the presented task, we conducted repeated-measures ANOVA with order and distance as within-subject variables and task as between-subject variable. Results showed significant effects for order, *F*(1, 111) = 19.25, *p* < .001, $\eta_{p}^{2}$ = .15. Ordered sequences (1,156 ms) were processed faster than non-ordered sequences (1,219 ms). Furthermore, a main effect of distance was found, *F*(1, 11) = 7.74, *p* = .006, $\eta_{p}^{2}$ = .06. Small distance sequences (1,204 ms) were processed slower than large distance sequences (1,172 ms). There was a significant interaction between order and distance, *F*(1, 113) = 10.51, *p* = .002, $\eta_{p}^{2}$ = .09. A significant effect of task was found, *F*(1, 113) = 6.64, *p* = .011, $\eta_{p}^{2}$ = .06, showing faster performance on the trials of the common order task (1,106 ms) compared with trials on the balanced order task (1,269 ms). Furthermore, interaction effects were found between order and task, *F*(1, 111) = 8.12, *p* = .005, $\eta_{p}^{2}$ = .07, and between distance and task, *F*(1, 111) = 4.44, *p* = .037, $\eta_{p}^{2}$ = .04. Together, these results show that the observed effects were modulated by the trial list composition of the tasks.

##### Modulation of presentation order

In the pre-registered analyses presented above, we did not take into account presentation order of the tasks. However, presentation order was part of our design and might have an important influence on how the different sequences are processed. Therefore, we conducted exploratory repeated-measures ANOVAs with presentation order as between-subject variable. Surprisingly, we found that presentation order played a role.

For the balanced order task, an effect of presentation order was observed, *F*(1, 55) = 7.03, *p* = .010, $\eta_{p}^{2}$ = .11, showing that the group that started with the common order task (1,157 ms) performed significantly faster on the balanced order task compared with the group that started with the balanced order task (1,378 ms).

For the common order task, no main effect of presentation order was observed, *F*(1, 56) = 3.43, *p* = .069, $\eta_{p}^{2}$ = .06. However, the repeated-measures ANOVA on all the trials of the common order task showed a significant interaction between distance and presentation order, *F*(1, 56) = 6.59, *p* = .013, $\eta_{p}^{2}$ = .11. Participants starting with the common order responded faster to large distance trials (1,185 ms) compared with small distance trials (1,198 ms), but this difference was not significant, *F*(1, 27) = 0.26, *p* = .614, $\eta_{p}^{2}$ = .01. Participants starting with the balanced order task responded significantly faster to large distance trials (978 ms) compared with small distance trials (1,074 ms), *F*(1, 29) = 30.32, *p* < .001, $\eta_{p}^{2}$ = .51.

A repeated-measures ANOVA on the ordered trials in the common order task also revealed a significant interaction between distance and presentation order, *F*(1, 56) = 6.28, *p* = .015, $\eta_{p}^{2}$ = .10. Participants starting with the common order task responded faster to small distance trials (1,100 ms) compared with large distance trials (1,161 ms) although this difference was not significant, *F*(27) = 4.16, *p* = .051, $\eta_{p}^{2}$ = .13. The participants starting with the balanced order task responded faster to large distance trials (983 ms) compared with small distance trials (1,023 ms), but this difference was also not significant, *F*(29) = 2.17, *p* = .151, $\eta_{p}^{2}$ = .07. In sum, these results show that presentation order modulated the general RT on the balanced order task and the observed effects in the common order task.

### Discussion

In the second experiment, we examined whether the absence of the RDE in the first experiment was due to the composition of the trial list. Therefore, participants performed two order tasks that differed in trial list composition. In both tasks, no RDEs were observed. In addition, results demonstrated that the observed effects were modulated by the trial list composition of the tasks. Furthermore, presentation order modulated the general reaction time for the balanced order task and the DEs for the common order task, that is, the task containing a large proportion of regularly ordered trials. These findings suggest that while the RDE has been considered as a robust effect in the order task (Lyons & Ansari, 2015; Lyons & Beilock, 2013), its appearance is actually highly dependent on the trial list composition and presentation order of tasks.

In contrast to the results observed in Experiment 1, no RDEs or DEs were observed for the balanced order task in Experiment 2. This difference could again be explained by the composition of the trial list. In Experiment 1, some of the ordered regular sequences with a medium and large distance (e.g., 2-4-6, 4-6-8, and 3-6-9, which resemble the multiplication table) might have been more familiar and could be more easily retrieved from memory than ordered regular sequences with a small distance (e.g., 3-4-5, 4-5-6, and 5-6-7). In Experiment 2, a larger variation of trials was presented. Among this larger variation of trials, there was probably an equal amount of familiar trials (that can be retrieved from long-term memory) for small and large distance sequences resulting in no difference in processing time.

In Experiment 2, we also further examined how different sequences were related to single digit addition and multiplication (i.e., arithmetic operations requiring retrieval) and multiple digit subtraction (i.e., an arithmetic operation requiring procedural strategies). Results showed that there were no significant differences regarding the associations of the sequences with the different arithmetic operations. There were also no differences between the order tasks regarding the size of the correlations between the sequences and arithmetic. While a specific category of sequences is probably primarily processed by a specific strategy, it might be the case that some sequences in a category are processed by an alternative strategy. For example, most ordered large distance sequences might be processed by comparison strategies (e.g., 1-3-5), but for some sequences also a retrieval strategy might be used (e.g., 2-4-6 because this sequence resembles the multiplication table of two). As a result, the correlations between the sequences and the arithmetic operations do not significantly differ across different arithmetic operations and do not differ between the two tasks. To gain more insight in the relation between specific sequences and certain arithmetic operations, future research could include conditions with merely sequences that elicit a specific strategy such as retrieval or comparison.

To sum up, performance on specific sequences in specific tasks did not correlate more strongly with certain arithmetic operations. Furthermore, the results indicate that the occurrence of the DEs is modulated by the trial list composition and the presentation order of the tasks. These results show that both the trial list composition and the presentation order of the tasks have a crucial influence on the strategies that are used when performing an order task.

## General discussion

Recently, there has been an increasing interest in the relation between ordinality and arithmetic performance, but the mechanisms underlying this relation remain relatively underspecified. The objective of the present study was to uncover these mechanisms by addressing two aims. First, we aimed to systematically unravel the strategies involved when processing different types of sequences in the order task. Therefore, we took all the possible dimensions of the sequences into account in the first experiment and examined the influence of the trial list composition in the second experiment. Second, we aimed to further examine how ordinality is related to arithmetic performance and mathematical reasoning.

In two experiments, we showed that observed behavioural effects in the order task are highly dependent on the trial list composition. In contrast to our hypothesis and previous observations, standard DEs were found for both ordered and non-ordered sequences in the first experiment, suggesting that participants did not use retrieval strategies. In the second experiment, we directly investigated whether the different compositions of the trial list in the first experiment—compared with previous studies—could explain why we did not replicate the finding of a RDE for ordered regular sequences. Two order tasks with a different composition of trials were presented, and the results showed that in both tasks no RDE was found. In both experiments, trials with the strongest associations (e.g., 1-2-3) were not present. Probably, the absence of these trials resulted in a weak reliance on retrieval strategies, and therefore no RDE was observed. Furthermore, results demonstrated that the observed effects were modulated by the trial list composition of the tasks. In addition, presentation order modulated the DEs for the task containing a large proportion of regularly ordered trials.

We believe that this is the first study that shows that the occurrence of the RDE is dependent on the trial list composition and presentation order of tasks. Two experiments demonstrated that the RDE does not occur when few retrieval trials are presented. Moreover, results revealed that the presentation order of the tasks plays a role for tasks containing numerous trials that elicit retrieval strategies. An implication of our findings is that researchers should be cautious when using the RDE as a predictor of arithmetic performance. This study demonstrates that the RDE is not as robust as previously thought and its presence is highly dependent on the trial list composition and the context in which the task is presented.

The finding that the cognitive strategy applied on a given trial can be influenced by the trial list composition in a given task is not new and has been reported earlier in several research domains of cognitive psychology, such as in the domains of problem solving (Luchins, 1942) and inhibition (Allport et al., 1994) and also in the domain of numerical cognition (Lemaire & Reder, 1999; Macizo & Herrera, 2011). For instance, Macizo and Herrera (2011) showed that an increased exposure to trials with a unit-decade incompatibility effect (i.e., an effect that occurs when the decade comparison leads to a different decision than the unit comparison) facilitated strategies that led to a correct response for these types of trials. From these findings, it was concluded that the processing of digits is modulated by cognitive control: participants adapt their strategies to the task demands. Similarly, Lemaire and Reder (1999) found that the trial list composition affected the parity effect in arithmetic verification tasks (i.e., better performance for false problems when there is a mismatch in the odd/even status of the presented answer and the correct answer). Results showed larger parity effects when there was a large proportion of false problems violating parity. Moreover, previous studies have not only shown that participants adapt their strategies to the task demands, but it has even been found that when a strategy is applied to a certain trial, it is more likely to be used on the next trial. For example, in a study by Lemaire and Lecacheur (2010), participants had to solve two digit addition problems. After being instructed which strategy to use for the first problem, they observed that participants showed a tendency to repeat the same strategy on consecutive trials. In the current study, we observed a similar influence of the trial list composition and presentation order.

A limitation of the current study is that we can only indirectly derive the strategies that are used from the behavioural effects. As a consequence, we can only derive the main strategy that is used for a specific category of sequences indirectly. To draw a more complete picture on the cognitive strategies that participants use when processing ordinality, future research would benefit from methods that would assess the applied strategies more directly. One possibility is to ask participants to perform the order task out loud. Furthermore, strategy reports could be used to gain more insight in the strategies that participants use.

Similar to previous studies, results in the current study also showed that ordinality was strongly related to arithmetic performance. Furthermore, the current study showed that there was a relation between ordinality and more advanced mathematical reasoning. However, this relation disappeared when fluid intelligence (measured with Raven’s APM test) was taken into account (Arthur & Day, 1994; Raven, 1938). The Raven is known to measure abstract reasoning skills and visuospatial reasoning (Raven, 1938; Waschl, 2017). Our observation that the relation between ordinality and mathematical reasoning was completely explained by fluid intelligence suggests that mathematical reasoning relies on higher order abstract reasoning skills. So far, findings about the relation between ordinality and more complex mathematics have been inconclusive. Morsanyi et al. (2018) concluded that ordinality uniquely predicts mathematical reasoning. An explanation for the inconsistency between the current study and the study of Morsanyi et al. (2018) is that different tasks were used to measure mathematical reasoning. Morsanyi et al. (2018) used the cognitive reflection task and probabilistic reasoning scale. The cognitive reflection task is known to measure inhibition and the probabilistic reasoning scale is known to measure statistical reasoning. It might be that specific aspects of mathematical reasoning such as inhibition and statistical reasoning are more uniquely related to ordinality compared with a more general mathematical reasoning measure that we included in our study and which covers a large range of mathematical reasoning problems. To pinpoint the relation between ordinality and mathematical reasoning, further research is necessary.

To conclude, the current study confirmed that ordinality is reliably related to arithmetic performance while the relationship between ordinality and mathematical reasoning appears to be completely explained by fluid intelligence. Ordinality is strongly related to arithmetic performance because it necessitates a constellation of different strategies that are themselves also important for arithmetic performance and they can be flexibly adapted to the given task demands.

## Supplemental Material

sj-docx-1-qjp-10.1177_17470218211016794 – Supplemental material for Ordinality: The importance of its trial list composition and examining its relation with adults’ arithmetic and mathematical reasoning Click here for additional data file. Supplemental material, sj-docx-1-qjp-10.1177_17470218211016794 for Ordinality: The importance of its trial list composition and examining its relation with adults’ arithmetic and mathematical reasoning by Helene Vos, Wim Gevers, Bert Reynvoet and Iro Xenidou-Dervou in Quarterly Journal of Experimental Psychology

## Notes

1. For ordered small, medium, and large distance trials, all small, medium, and large distance ordered sequences were taken into account.

2. A mistake was made in the pre-registration. The amount of trials that were pre-registered for the arithmetic verification tasks (i.e., 52 trials) deviate from the amount of trials that were administered (i.e., 48 trials).
