# Supplementary material for: Ordinality: The importance of its trial list composition and examining its relation with adults’ arithmetic and mathematical reasoning
Source: Q J Exp Psychol (Hove). 2021 May 24;74(11):1935–52. doi: 10.1177/17470218211016794 (PMC8450998; doi:10.1177/17470218211016794)
Supplement: sj-docx-1-qjp-10.1177_17470218211016794 – Supplemental material for Ordinality: The importance of its trial list composition and examining its relation with adults’ arithmetic and mathematical reasoning [file sj-docx-1-qjp-10.1177_17470218211016794.docx]

SUPPLEMENTARY MATERIALS for:

**Ordinality: The importance of its trial list composition and examining its relation with adults’ arithmetic and mathematical reasoning**

Helene Vos, Wim Gevers, Bert Reynvoet & Iro Xenidou-Dervou

**Supplementary Material A: By-item linear regression in Experiment 1**

**Dimensions driving ordinality**

***Reaction times.***

To examine which dimensions drive the RT on the order task, we conducted by-item linear regressions with the dimensions of the order task as predictors and median RT for correct responses on the order task as dependent variable. Firstly, we conducted a by-item linear regression for both ordered^[[1]](#footnote-1)^ and non-ordered trials with order and distance as predictors. Secondly, because ordered trials can also be distinguished regarding regularity and direction, we conducted a by-item linear regression for the ordered trials separately with direction, regularity and distance as predictors.

Results on the first by-item linear regression on all the trials (Table 1) showed that the model was predictive for the median RT on the order task, *F*(2, 14786) = 42.64, *p* <.001, *R*^2^ = .01. Order and distance were both unique predictors. Ordered sequences were processed faster than non-ordered sequences and large distance sequences were processed faster than small distance sequences.

Table 1. *Multiple regression with the median RT of the order task as dependent variable and order and distance as independent variables.*

| Independent variables | Standardized *β* | *t* | *p* |  |
| --- | --- | --- | --- | --- |
| Order  Distance | .06  -.04 | 7.78  -5.01 | <.001  <.001 |  |

*R***^2^ =** .01**

* *p* < .05, ** *p* < .01

Subsequently, we investigated which dimensions of the ordered sequences (i.e. distance, regularity and direction) predicted the median RT on the order task (Table 2). Again, we conducted a by-item linear regression with the median RT on the order task as dependent variable and direction, regularity and distance as predictors. The median RT on the order task was predicted by the model, *F* (3, 7217) = 11.80, *p* < .001, *R*^2^ = .01. Direction, distance and regularity predicted the median RT on the order task. Large distance sequences were processed faster than small distance sequences. Regular sequences were processed faster than irregular sequences. Ascending sequences were processed faster than descending sequences.

| Independent variables | Standardized *β* | *t* | *p* |  |
| --- | --- | --- | --- | --- |
| Direction  Regularity  Distance | .03  .04  -.05 | 2.24  3.71  -4.11 | .025  <.001  <.001 |  |

Table 2. *Multiple regression with the median RT of the order task as dependent variable and direction, regularity and distance as independent variables.*

*R***^2 =^** .01**

* *p* < .05, ** *p* < .01

***Accuracies.*** Results of the by-item linear regression on all the trials (Table 3) revealed that the model was predictive for the accuracy score on the order task, *F*(2, 16413) = 67.99, *p* <.001, *R*^2^ = .01. Order and distance were both unique predictors.

| Independent variables | Standardized *β* | *t* | *p* |  |
| --- | --- | --- | --- | --- |
| Order  Distance | .07  .06 | 9.10  7.29 | <.001  <.001 |  |

Table 3. *Multiple regression with the accuracy score of the order task as dependent variable and order and distance as independent variables.*

*R***^2^ =** .01**

* *p* < .05, ** *p* < .01

A by-item linear regression on the ordered trials (Table 4) showed that the accuracy score on the order task was predicted by the model, *F* (3, 8204) = 42.44, *p* < .001, *R*^2^ = .02. Direction, distance and regularity were unique predictors.

| Independent variables | Standardized *β* | *t* | *p* |  |
| --- | --- | --- | --- | --- |
| Direction  Regularity  Distance | -.08  -.09  .04 | -7.15  -7.90  3.73 | <.001  <.001  <.001 |  |

Table 4. *Multiple regression with the accuracy of the order task as dependent variable and direction, regularity and distance as independent variables.*

*R***^2 =^** .02**

* *p* < .05, ** *p* < .01

**Supplementary Material B: Accuracies for the repeated-measures ANOVA in Experiment 1**

**The effects of order, distance, regularity and direction**

To examine the influence of order and distance, we conducted a 2 (order: order, non-order) x 3 (distance: small, medium, large) repeated measures ANOVA. A main effect of order was found, *F*(1, 56) = 5.53, *p* = .022, *η^2^_p_* = .09. Non-ordered sequences were processed more accurately than ordered sequences. Additionally, a main effect of distance was observed, *F*(1.58, 88.58) = 25.06, *p* <.001, *η^2^_p_* = .31. Sequences with large distance were processed more accurately than sequences with a medium distance and sequences with a medium distance were processed more accurately than sequences with a small distance. Finally, results indicated no interaction between order and distance, *F*(1.69, 94.68) = 1.32, *p* =.270, *η^2^_p_* = .02.

For ordered sequences, we examined the relation between direction, regularity and distance by a 2 (direction: ascending, descending) x 2 (regularity: regular, irregular) x 3 (distance: small, medium, large) repeated measures ANOVA. Results showed a main effect of direction, *F*(1, 56) = 7.79, *p* = .007, *η^2^_p_* = .12 and a main effect of distance, *F*(1.81, 101.37) = 8.14, *p* = .001, *η^2^_p_* = .13. Ascending sequences were processed more accurately than descending sequences. Sequences with large distance were processed more accurately than sequences with a medium distance and sequences with a medium distance were processed more accurately than sequences with a small distance. An effect of regularity was found, *F*(1, 56) = 7.66, *p* = .008, *η^2^_p_* = .12. Regular sequences were processed more accurately than irregular sequences. The interaction between direction and regularity, was significant *F*(1, 56) = 5.24, *p* = .026, *η^2^_p_* = .09. There was an interaction between direction and distance, *F*(2, 112) = 4.98, *p* = .008, *η^2^_p_* = .08. Furthermore, an interaction between regularity and distance was observed, *F*(2, 112) = 3.49, *p* = .034, *η^2^_p_* = .06. Finally, there was a three-way interaction between direction, regularity and distance, *F*(2, 112) = 4.91, *p* = .009, *η^2^_p_* = .08.

**Supplementary Material C: Accuracies for the repeated-measures ANOVA in Experiment 2**

**Common order task**

***Accuracies.*** The influence of order and distance was investigated by 2 (order: order, non-order) x 2 (distance: small, large) repeated measures ANOVA. Results demonstrated no effects of order, *F*(1, 57) = .79, *p* < .377, *η^2^_p_* = .01 and distance, *F*(1, 57) = 2.70, *p* = .106, *η^2^_p_* = .05. Additionally, an interaction between order and distance was found, *F*(1, 57) = 9.52, *p* = .003, *η^2^_p_* = .14. For the ordered sequences, no distance effect was observed, *t*(57) = 1.00, *p* = .323. For non-ordered sequences, a standard distance effect was found, *t*(57) = -3.63, *p* = .001.

For the ordered trials, we examined the influence of direction and distance by 2 (direction: ascending, descending) x 2 (distance: small, large) repeated measures ANOVA. A main effect of direction was found, *F*(1, 57) = 31.35, *p* < .001, *η^2^_p_* = .36. Ascending sequences were processed more accurately than descending sequences. No effect of distance was found, *F*(1, 57) = 1.06, *p* = .307, *η^2^_p_* = .02. Finally, no interaction between direction and distance was observed, *F*(1, 57) = 1.55, *p* = .219, *η^2^_p_* = .026

**Balanced order task**

***Accuracies***. The influence of order and distance on the accuracy of the order was investigated by 2 (order: order, non-order) x 2 (distance: small, large) repeated measures ANOVA. No effect of order was observed, *F*(1, 56) = 1.73, *p* = .194, *η^2^_p_* = .03. Results showed a significant effect of distance, *F*(1, 56) = 11.57, *p* = .001, *η^2^_p_* = .17. Large distance sequences were processed more accurately than small distance sequences. No interaction was found between order and distance, *F*(1, 56) = .35, *p* = .555, *η^2^_p_* = .01.

For the ordered trials, we examined the influence of direction, regularity and distance by 2 (direction: ascending, descending) x 2 (regularity: regular, irregular) x 2 (distance: small, large) repeated measures ANOVA. Results showed a significant effect of direction, *F*(1, 56) = 5.10, *p* = .028, *η^2^_p_* = .08. Ascending sequences were processed more accurately than descending sequences. A significant effect of distance was observed, *F*(1, 56) = 7.33, *p* = .009, *η^2^_p_* = .12 Large distance sequences were processed more accurately than descending sequences. No effect of regularity was found, *F*(1, 56) = 2.86, *p* = .097, *η^2^_p_* = .05. No interaction effects were found.

**Supplementary Material D: Repeated-measures ANOVA in Experiment 2 with presentation order as between-subjects variable**

**Common Order task**

***Reaction Times.*** The influence of order and distance on the median RT for correct responses was examined by a 2 (order: order, non-order) x 2 (distance: small, large) repeated-measures ANOVA with presentation order as between subject-variable. A significant effect of order was found, *F*(1, 56) = 20.07, *p <* .001, *η^2^_p_* = .26. Ordered sequences were processed faster compared to non-ordered sequences. Results showed an effect of distance, *F*(1, 56) = 11.83, *p=* .001, *η^2^_p_* = .17 and a significant interaction between order and distance, *F*(1, 56) = 14.55 *p <* .001, *η^2^_p_* = .21. Results indicated no effect of presentation order, *F*(1, 56) = 3.43, *p* = .069, *η^2^_p_* = .06. Finally, an interaction effect between distance and presentation order was observed, *F*(1, 56) = 6.59, *p=* .013, *η^2^_p_* = .11. Participants starting with the common order responded faster to large distance trials (1185 ms) compared to small distance trials (1198 ms), but this difference was not significant, *F*(1, 27) = .26, *p* = .614, *η^2^_p_* = .01. Participants starting with the balanced order task responded significantly faster to large distance trials (978 ms) compared to small distance trials (1074 ms), *F*(1, 29) = 30.32, *p* < .001, *η^2^_p_* = .51.

For the ordered trials, the influence of direction and distance on the median RT for correct responses was investigated by a 2 (direction: ascending, descending) x 2 (distance: small, large) repeated measures ANOVA with presentation order as between-subject variable . Results showed a significant effect of direction, *F*(1, 56) = 52.85, *p <* .001, *η^2^_p_* = .49. There was no effect of distance, *F*(1, 56) = .27, *p* = .605, *η^2^_p_* = .01, no interaction between distance and direction, *F*(1, 56) = 1.29, *p* = .261, *η^2^_p_* = .02 and no effect of presentation order, *F*(1, 56) = 2.21, *p* = .142, *η^2^_p_* = .04. Again a significant interaction effect was found between distance and presentation order, *F*(1, 56) = 6.28, *p =* .015, *η^2^_p_* = .10. Participants starting with the common order task responded faster to small distance trials (1100 ms) compared to large distance trials (1161 ms) although this difference was not significant *F*(27) = 4.16, *p* = .051, *η^2^_p_* = .13. The participants starting with the balanced order task responded significantly faster to large distance trials (983 ms) compared to small distance trials (1023 ms), but this difference was also not significant, *F*(29) = 2.17, *p* = .151, *η^2^_p_* = .07.

***Accuracies.*** To investigate the influence of order and distance, we conducted a 2 (order: order, non-order) x 3 (distance: small, large) repeated measures ANOVA. Results showed no effect of order, *F*(1, 56) = .72, *p* = .400, *η^2^_p_* = .01 and no effect of distance, *F*(1, 56) = 2.59, *p* = .113, *η^2^_p ._*= .04. An interaction effect between order and distance was found, *F*(1, 56) = 10.34, *p* =.002, *η^2^_p_* = .16. For the ordered sequences, no distance effect was found, *t*(57) = 1.00, *p* = .323. For non-ordered sequences, a standard distance effect was observed, *t*(57) = -3.63, *p* = .001. No effect of presentation order was observed, *F*(1, 56) = .27, *p* = .603, *η^2^_p_* = .01 and no interaction effects with presentation order were found.

For the ordered trials, we conducted a 2 (direction: ascending) x 2 (distance: small, large) repeated measures ANOVA. A significant effect of direction was found, *F*(1, 56) = 31.96, *p* < .001, *η^2^_p_* = .36. No effect of distance was observed, *F*(1, 56) = 1.22, *p* = .274, *η^2^_p_* = .02. Furthermore, results showed no interaction between distance and direction, *F*(1, 56) = 1.49, *p* = .228, *η^2^_p_* = .03. No effect of presentation order was found, *F*(1, 56) = .25, *p* = .620, *η^2^_p_* = .004 and there were no interaction effects with presentation order.

**Balanced Order task**

***Reaction times.*** We investigated the role of order and distance on the median RT for correct responses by a 2 (order: order, non-order) x 2 (distance: small, large) repeated measures ANOVA with presentation order as between-subject variable. Results showed no effect of order, *F*(1, 55) = 1.79, *p* = .187, *η^2^_p_* = .03. Similarly, no effect of distance was observed, *F*(1, 55) =.23, *p* = .64, *η^2^_p_* = .004. Also the interaction between order and distance was not significant, *F*(1, 55) = .80, *p* = .376, *η^2^_p_*  = .01. An effect of presentation order was observed, *F*(1,55) = 7.03, *p* = .010, *η^2^_p_* = .11, but no interaction effects with presentation order were found. The group that started with the common order task performed significantly faster on the task compared to the group that started with the balanced order task.

For the ordered trials we examined the influence of direction, regularity and distance on the median RT for correct responses by conducting a 2 (direction: ascending, descending) x 2 (regularity: regular, irregular) x 2 (distance: small, large) repeated measures ANOVA with presentation order as between-subjects-variable. Results showed a significant effect of regularity, *F*(1, 54) = 8.39, *p* = .005, *η^2^_p_* = .13. There was a significant effect of direction, *F*(1, 54) = 4.37, *p* = .041, *η^2^_p_* = .08. Ascending sequences were processed faster than descending sequences. Regular sequences were processed faster than irregular sequences. No effect of distance was found, *F*(1, 54) = .40, *p* = .531, *η^2^_p_* = .01. An effect of presentation order was observed, *F*(1, 54) = 4.66, *p* = .035, *η^2^_p_* = .08. Furthermore, no interaction effects were observed.

***Accuracies.*** To examine the influence of order and distance, a 2 (order: order, non-order) x 3 (distance: small, large) repeated measures ANOVA was performed. A significant effect of distance was found, *F*(1, 55) = 11.59, *p* = .001, *η^2^_p_* = .17. Large distance sequences were processed more accurately than small distance sequences. Results showed no effect for order, *F*(1,55) = 1.68, *p* = .200, *η^2^_p_* = .030 and no interaction between order and distance, *F*(1, 55) = .33, *p* = .565, *η^2^_p_* = .01. Finally, results demonstrated no effect of presentation order, *F*(1, 55) = .12, *p* = .736, *η^2^_p_* = .002 and no interaction effects with presentation order.

For the ordered trials, we conducted a 2 (direction: ascending) x 2 (regularity: regular, irregular) x 2 (distance: small, large) repeated measures ANOVA. Results demonstrated a significant effect of direction, *F*(1, 55) = 5.07, *p* = .028, *η^2^_p_* = .08 and a significant effect of distance, *F*(1,55) = 7.20, *p* = .010, *η^2^_p_* = .12. Ascending sequences were processed more accurately than descending sequences. Large distance sequences were processed more accurately than small distance sequences. No effect of regularity was found, *F*(1, 55) = 2.85, *p* = .097, *η^2^_p_* = .05. Furthermore, no interaction effects were found. Finally results showed no effect of presentation order, *F*(1,55) = .31, *p* = .583, *η^2^_p_* = .01 and no interaction effects with presentation order.

1. In this analysis, ordered small distance trials included ascending regular small distance, ascending irregular small distance, descending regular small distance and descending irregular small distance. Similarly, for ordered medium and large distance trials, all medium and large distance ordered sequences were taken into account. [↑](#footnote-ref-1)
